# T cells in chronic lymphocytic leukemia: can they fight?

**DOI:** 10.18632/oncotarget.22277

**Published:** 2017-11-03

**Authors:** Anna Vardi, Kostas Stamatopoulos, Anastasia Hadzidimitriou

**Affiliations:** Kostas Stamatopoulos: Institute of Applied Biosciences, Centre for Research and Technology Hellas, Thessaloniki, Greece and Department of Immunology, Genetics and Pathology, Uppsala University, Uppsala, Sweden

**Keywords:** T cell repertoire, CLL, NGS immunoprofiling

Chronic lymphocytic leukemia (CLL) is a malignancy of mature B cells critically dependent on microenvironmental stimulation for their survival and proliferation. For decades, research focused on the neoplastic B cells, revealing the central role of the B cell receptor (BcR) in CLL pathophysiology and leading to the recent development of drugs that exhibit remarkable clinical efficacy by inhibiting B cell signalling, critically including the BcR pathway. However, besides transducing signals provided through antigenic stimulation, the BcR molecule also serves as a channel of CLL cell communication with cognate T cells. Functional studies of the immune synapse in CLL have unveiled an intricate crosstalk of CLL B cells with T cells, culminating in the induction of T-cell anergy, thus allowing the neoplastic clone to escape immune surveillance [1, 2]. Moreover, studies in animal models have demonstrated that CLL clones depend on trophic signals provided by T cells [3].

That notwithstanding, little is known regarding the architecture of the T-cell repertoire in CLL. To address this knowledge gap and to further dwell into preliminary findings obtained by low-throughput techniques suggesting antigenic selection [4], we conducted a large-scale, next-generation sequencing (NGS) study of the T-cell receptor beta chain (TRB) gene repertoire in 57 CLL patients [5]. The study focused on cases assigned to well-characterized subsets with stereotyped clonotypic BcR immunoglobulin (IG), thus most evidently selected by antigens, but also included CLL patients carrying non-subset BcR IG rearrangements, so as to explore T-cell immunogenetic features that can be common across all CLL.

Our study revealed the restricted nature but also the dynamics of the T cell repertoire in CLL, with clonotypes that persist and expand over time, thus alluding to persistent antigenic stimulation. An important finding concerned the existence of shared clonotypes amongst different patients, especially those belonging to the same stereotyped subset. Moreover, comparison to public databases indicates that these clonotypes may be disease-specific, altogether corroborating that antigenic elements may be selecting T cells in a CLL subset-specific context. On these grounds, we hypothesized that these could be the same antigens selecting the malignant clone or tumor-derived antigens. Recent evidence of homotypic BcR interactions underlying malignant B cell selection in CLL may in fact unify these scenarios into one, where epitopes expressed on the clonotypic BcR IG are recognized by both malignant B cells, triggering downstream BcR signaling, as well as cognate T cells, which clonally expand but somehow fail to mount a truly effective anti-tumor response [6] (Figure 1).

**Figure 1:**
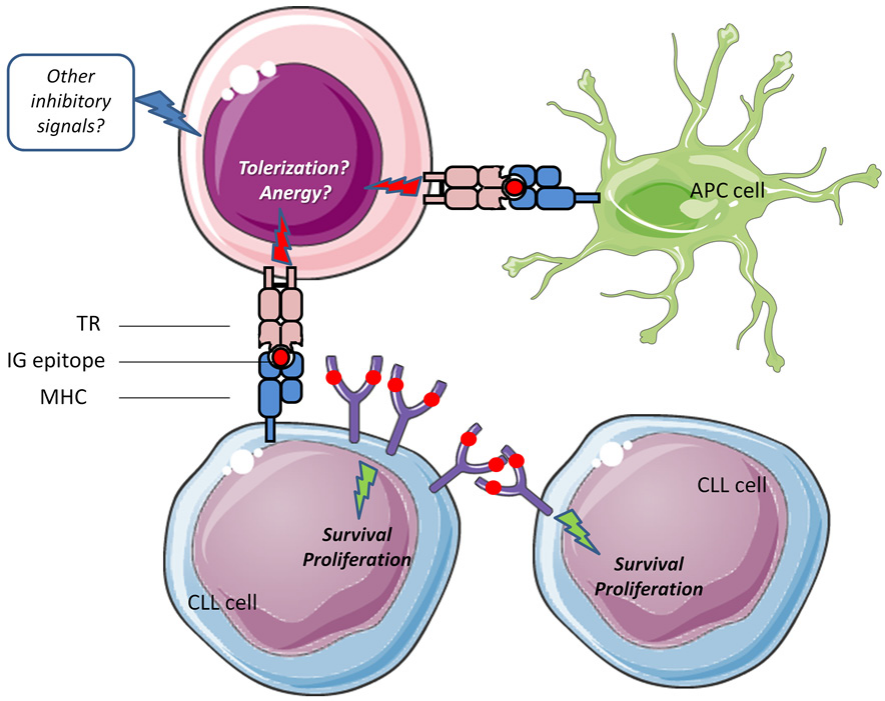
Schematic representation of a scenario where an epitope (red dot) expressed on the clonotypic BcR IG is recognized by: (i) CLL cells through homotypic interactions, triggering pro-malignant downstream BcR signaling, and (ii) cognate T cells through presentation by CLL cells or other antigen-presenting cells (APCs) This type of T-cell stimulation may, together with other inhibitory signals from the microenvironment, drive T-cells towards an anergic state, thereby facilitating tumor escape from immune surveillance.

This information can prove clinically relevant in the era of new drugs designed to stimulate a sustained anti-tumor effect via the host immune system rather than directly target the malignant cells, such as immunomodulatory drugs acting on the level of B-T cell synapse and immune checkpoint inhibitors. In fact, lenalidomide has recently been tested in CLL and demonstrated clinical benefit [7]. That notwithstanding, recent evidence supports that BcR inhibitors, besides directly targeting CLL B cells, may also be interfering with T cell effectors. In particular, the BTK inhibitor ibrutinib has been shown to boost the CD8^+^ cytotoxic population. Moreover, the PI3Kδ inhibitor idelalisib may directly affect T-cell cytokine production, reducing inflammatory cytokines such as CD40L. In addition, BcR inhibitors may impact on T cells indirectly by interfering with the ability of the malignant B cells to induce T cell tolerance. Hence, arguably, novel drugs may exert anti-tumor action also through effects on the CLL T cells.

Little information exists on the dynamics of TR repertoire in CLL as a result of treatment, especially with novel agents. In particular, the single available publication involves only ibrutinib and reports that ibrutinib therapy increases diversification of the T cell compartment in CLL patients, contributing to cellular immune reconstitution [8]. Our group is currently investigating the impact of BcR signaling inhibitors versus standard chemoimmunotherapy on CLL T cells by longitudinal, pre- and post-treatment profiling of the TR repertoire using NGS. This is anticipated to offer insight into the mechanism of action of novel drugs/drug combinations utilized for CLL treatment, particularly their impact on CLL microenvironment, and ultimately aims to reveal immune signatures either pre- or post-treatment that may serve as predictors of response, thereby assisting in implementing personalized treatment approaches. Preliminary findings show that chemoimmunotherapy increases T cell clonality likely through an ablative mechanism, in contrast to signaling inhibitors which retain T cell clones that may have developed in response to tumor antigens and possibly activate them, with obvious clinical implications. Needless to say, these observations have to be complemented with functional studies, confirming the impact of treatment to the effector properties of T cells.

Overall, T cells emerge as a critical player in CLL pathophysiology and have thus become an enticing target, which actually coincides with the development of novel drugs that harness the lymphoma microenvironment. In-depth immunoprofiling of the T cell repertoire, paired with functional characterization, may reveal mechanisms to abrogate T cell tolerance and translate directly into clinical practice, especially in the context of boosting the host immune surveillance for sustained MRD eradication.

## References

[1] Görgün G (2005). J Clin Invest.

[2] Ramsay AG (2013). Blood.

[3] Bagnara D (2011). Blood.

[4] Vardi A (2016). Clin Cancer Res.

[5] Vardi A (2017). Leukemia.

[6] Minici C (2017). Nat Commun.

[7] Shanafelt TD (2013). Blood.

[8] Yin Q (2017). J Immunol.

